# Pd Nanoparticles Supported on Tubular g-C_3_N_4_: Enhanced Selectivity in Acetylene Double Carbonylation

**DOI:** 10.3390/ma19173673

**Published:** 2026-08-29

**Authors:** Caimei Ma, Jiangbing Li, Xinlu Fan, Qingyang Yu

**Affiliations:** 1 College of Chemical and Environmental Engineering, Xinjiang Shihezi Vocational Technical College, Shihezi 832000, China; mcm5120@163.com (C.M.); fxl239756@gmail.com (X.F.); 2 School of Chemistry and Chemical Engineering, Shihezi University, Shihezi 832000, China; lature00@gmail.com

**Keywords:** acetylene dicarbonylation, Pd-based catalyst, tubular g-C_3_N_4_, carbon defect structure

## Abstract

Aiming at the problem that the selectivity of heterogeneous catalysts is difficult to be controlled and the active components are easy to be lost in the acetylene double carbonylation reaction, this paper constructs a highly efficient Pd-based catalyst through the combination of carrier morphology engineering and surface basicity regulation. Using melamine/urea as a precursor, tubular carbon nitride (TCN) was constructed by hydrothermal calcination, and then Pd/TCN-series catalysts were prepared by ultrasonic-assisted impregnation loading 5 wt% Pd and heat treatment in a N_2_ atmosphere. At the same time, bulk g-C_3_N_4_ (BCN) and activated carbon (AC), TiO_2_, and ZSM-5 supported systems were used as controls. The characterization results show that compared to BCN, TCN-500 (calcined at 500 °C) has a higher specific surface area (40.19 m^2^/g vs. 12.47 m^2^/g) and pore volume (0.1850 cm^3^/g vs. 0.0792 cm^3^/g), which provides abundant anchoring sites and efficient mass-transfer channels for Pd nanoparticles. After the introduction of Pd, the total base amount of the catalyst increased significantly from 0.4866 to 1.7172 mmol/g, and the strong Lewis-base center was significantly enhanced. TEM/XRD confirmed that Pd was uniformly dispersed on TCN-500 and mainly exposed the (111) crystal plane. XPS further revealed that there was a stronger electron-coupling effect between Pd and the support. Under the reaction conditions of 70 °C and 5 h, the selectivity of Pd/TCN-500 to dimethyl butenedioate was up to 83.7% (acetylene conversion was 59.6%), which was significantly better than that of the contrast carrier system. The cycle test showed that the selectivity and conversion of the catalyst were reduced to 54.4% and 41.1%, respectively, on the third use. The performance degradation was mainly attributed to the oxidative damage of the TCN nanotube skeleton and the passivation of the surface active defect sites. In this study, the synergistic effect of tubular morphology and surface Lewis basicity effectively stabilized the Pd active center and regulated the product selectivity, which provided a new idea for the development of efficient heterogeneous catalysts for acetylene double carbonylation in non-petroleum routes.

## 1. Introduction

Unsaturated dicarboxylate esters, such as dimethyl maleate and dimethyl fumarate and their derivatives, can be efficiently synthesized by acetylene double carbonylation with carbon monoxide as the carbonyl source, which has been applied in the field of fine chemicals [1]. The existing catalytic systems are mostly concentrated in the homogeneous path, and the transition metals Pd, Rh, Ru, and Ir are widely used in carbonylation reactions. Among them, the system with palladium salt as the active center has both high activity and selectivity. Classical studies have shown that the PdCl_2_-FeCl_3_-H_2_SO_4_ catalytic system constructed by Xu et al. in 1983 can convert acetylene into (cis, trans) dibutyl butenedioate under normal pressure and 60 °C with butanol as a solvent [2]. The PdBr_2_-LiBr-CH_3_CN catalytic system constructed by Bruk et al. in 1995 was used to synthesize succinic anhydride by the carbonylation of acetylene at 40 °C under atmospheric pressure, with a yield of about 70% [3]. In terms of mechanisms, acetylene dicarbonylation usually involves (i) a reversible valence cycle of Pd^2+^/Pd^0^; (ii) co-catalysts such as iron halide, copper halide, and KI, which reoxidize the deactivated Pd^0^ to Pd^2+^; and (iii) air (or O_2_) that is used as the terminal oxidant to regenerate the reduced cocatalyst. Although the homogeneous system can improve the yield and selectivity through the molecular-level contact area, it still faces engineering bottlenecks such as difficult separation of catalyst and product, difficult recovery and a complex subsequent purification process [4]. In contrast, the heterogeneous catalysts for acetylene double carbonylation usually exist in solid form, which has the advantages of easy phase separation from the reaction system and convenient catalyst recovery and reuse. The active metal or active site is fixed on the carrier, which helps to reduce metal leaching and ligand loss, reduce metal residues in the product, and improve the environmental friendliness and safety of the process. Heterogeneous catalysts can achieve fine regulation of activity and selectivity by regulating the pore structure, surface acidity and alkalinity, and metal dispersion state of the support and have great potential for application in green chemical and industrial catalytic processes. Among them, the palladium-based heterogeneous system can obtain double carbonylation products under mild conditions and exhibit better overall performance. It has been reported that Pd/α-Fe_3_O_4_ supported on α-Fe_3_O_4_ can reduce the poisoning of CO in Pd, enhance the adsorption/activation of CO, and make acetylene attack the Pd-CO bond. Under the condition of <80 °C, the conversion of acetylene is 82.5% and the selectivity of dimethyl fumarate is 75.6% [5]. The CoO_x_-Pd/HAC catalyst prepared by the strategy of in situ formation of Pd nanoparticles has the best activity when 3% Co is doped, with acetylene conversion of 75.4% and dimethyl maleate selectivity of 86.2% [6]. Other supported Pd catalysts (such as PdCl_2_/SiO_2_ and Pd/AC) have also shown good activity and stability [7,8]. In summary, the rational design of Pd-based heterogeneous catalysts around the regulation of carrier structure and metal dispersion is of great significance to the efficient and green conversion of acetylene dicarbonylation.

The structure and chemical stability of the catalyst support and the existence form and interface interaction of the active metal on the support are the key to determining the catalytic effect. Among them, a g-C_3_N_4_ material with a two-dimensional stacking structure and π-conjugated planar layer has the advantages of a simple synthesis method, good chemical and thermal stability and a low production cost. However, its commonly layered or blocky morphology leads to the limitation of specific surface area and accessible sites, as well as the obstruction of metal dispersion and mass transfer, which limits its wide applications as a supported catalyst carrier. In contrast, tubular carbon nitride (TCN) has attracted extensive attention in the field of catalysis due to its hollow, tubular skeleton structure and abundant active surface sites [9]. Tubular g-C_3_N_4_ based on a triazine skeleton has a high density of nitrogen-containing functional groups, which can be used as the coordination and anchoring sites of metal species. On one hand, the electron transfer between noble metals and carbon nitride and the strong interfacial interaction help to enhance the stability of the metal active center; on the other hand, the spatial confinement effect and geometric constraints brought by the nanotube structure can reduce the mass transfer resistance and effectively prevent the loss and sintering of metal particles [10]. In addition, structural defects such as carbon vacancies can modulate the electronic structure and redox capacity of the carrier, providing metal capture sites, thereby simultaneously improving the metal dispersion and interfacial reaction microenvironment and synergistically improving the catalytic activity and stability [11]. The tubular g-C_3_N_4_ has been extensively studied as a metal catalyst carrier in carbonylation reactions [12,13,14,15,16].

Based on this, in this study, carbon-defect tubular carbon nitride was prepared by the hydrothermal method combined with high-temperature treatment, and Pd nanoparticles were loaded by the impregnation method to prepare a Pd/TCN heterogeneous catalyst. The purpose of this study is to reveal the regulation of the carbon-defect structure and tubular morphology on the dispersion and electronic environment of Pd species and to systematically evaluate their catalytic performance in the acetylene dicarbonylation reaction. This work is expected to provide new ideas for acetylene conversion in non-petroleum routes and provide a theoretical and experimental basis for the design of efficient and stable green catalysts.

## 2. Experimental Method

### 2.1. Experimental Materials and Instruments

Experimental materials melamine, urea, palladium chloride, potassium iodide, TiO_2_, and ZSM-5 were analytically pure (Shanghai, China). Activated carbon (≥100 mesh) and methanol (chromatographic HPLC-grade) were purchased from the official website of Aladdin Reagent (Shanghai, China). High-purity acetylene (99.99%), CO (99.99%) and N_2_ (99.99%) were used as gases.

The experimental instruments were as follows: a high-pressure reactor (HT-250FJ-C), Shanghai Huotong Experimental Instrument Co., Ltd. (Shanghai, China); a tubular furnace (TL1200), Nanjing Boyuntong Instrument Technology Co., Ltd. (Nanjing, China); a blow dryer (DHG-9140A), Shanghai Qixin Scientific Instrument Co., Ltd. (Shanghai, China); a gas chromatograph (GC-2014), Shanghai Shimadzu Experimental Equipment Co., Ltd. (Shanghai, China); and high-performance liquid chromatography (HPLC) (VH-C18-A), Thermo Fisher Scientific (China) Co., Ltd., (Shanghai, China).

### 2.2. Preparation of Catalyst

#### 2.2.1. Synthesis of Carbon Nitride Carrier

Preparation of tubular carbon nitride (TCN): firstly, 1.43 g urea and 1.0 g melamine were dissolved in 60 mL deionized water and stirred at 90 °C for 10 min. After complete dissolution, this was transferred to a high-pressure hydrothermal kettle and treated with different hydrothermal temperatures and times. After cooling, the product was collected, centrifuged at 8000 r/min for 5 min, washed with deionized water to neutralize, and dried in an oven at 60 °C for 10 h to obtain a carbon–nitrogen complex (CNC-X-Y). Subsequently, TCN-400, TCN-500 and TCN-600 were obtained by calcining at 400, 500 or 600 °C for 4 h under a nitrogen atmosphere at 5 °C/min. In the comparative experiment, bulk g-C_3_N_4_ (BCN) was prepared by direct pyrolysis of melamine (600 °C, 2 h).

#### 2.2.2. Preparation of Pd-Based Catalysts

(1) Preparation of PdCl_2_ solution

A total of 100 mg PdCl_2_ was dissolved in 0.5 mL concentrated hydrochloric acid (36~38%), added to deionized water, and stirred at 70 °C for 0.5 h in a fume hood. After cooling, the chloropalladium acid solution was fixed in a 100 mL brown volumetric flask, sealed and stored, and then used.

(2) Preparation of catalyst with theoretical palladium loading of 5%

The 500 mg support was dispersed in 30 mL deionized water and sonicated for 30 min, and 42 mL PdCl_2_ solution was added. After ultrasonication for 1 h and stirring at room temperature for 24 h, it was finally dried at 70 °C for 10 h. Pd/TCN-400, Pd/TCN-500 and Pd/TCN-600 were prepared by calcination at 400 °C, 500 °C and 600 °C for 4 h in a nitrogen atmosphere, respectively. For comparison, Pd/BCN, Pd/AC, Pd/TiO_2_ and Pd/ZSM-5 were synthesized by the same method.

### 2.3. Catalyst Performance Evaluation

A total of 30 mg of catalyst was added to the reaction system, with 30 mL of methanol as a solvent and 100 mg of KI as a promoter, and placed in a high-pressure reactor. After confirmation that the reaction kettle was well-sealed, 17.08 mmol of acetylene was slowly introduced and pre-stirred for 30 min under mechanical stirring conditions until the pressure in the kettle was basically stable to ensure that the acetylene was fully dissolved in the solution. Subsequently, CO was introduced to adjust the reaction pressure to 1.6 MPa, and then air was introduced to increase the total pressure to 4.8 MPa. The heating device was opened, the reaction temperature was controlled at 70 °C, the mechanical stirring speed was set to 800 r/min, and the reaction was carried out for 5 h under the above conditions. After the reaction was completed, the heating was stopped and the reaction system was naturally cooled to room temperature. The reaction products were separated by centrifugation, and the gas phase and liquid phase were qualitatively and quantitatively analyzed by gas chromatography (GC-2014C) (Shimadzu, Kyoto, Japan) and high-performance liquid chromatography (VH-C18-A) (Thermo Fisher, Waltham, MA, USA), respectively. The catalytic performance was characterized by acetylene conversion (X) and the selectivity of target product dimethyl butenedioate (S).

Conversion of acetylene (X):

(1) $$X\left(\%\right)=\frac{n_{0\left(C_{2}H_{2}\right)}-n_{1\left(C_{2}H_{2}\right)}}{n_{0\left(C_{2}H_{2}\right)}}$$

Selectivity of dimethyl butenedioate (S):

(2) $$S\left(\%\right)=\frac{n_{2}}{n_{0\left(C_{2}H_{2}\right)}-n_{1\left(C_{2}H_{2}\right)}}$$

In these formulae, n_0_ (C_2_H_2_) is the amount of acetylene in the high-pressure reactor, in mol; n_1_(C_2_H_2_) represents the remaining amount of acetylene, in mol; and n_2_ is the amount of dimethyl butenedioate obtained after the reaction, in mol.

### 2.4. Material Characterization

The morphology of the catalyst was analyzed by SEM (Hitachi, Tokyo, Japan) and HRTEM (JEOL, Tokyo, Japan); “Scanning electron microscopy (SEM) analysis was performed using a ZEISS Sigma 300 field-emission scanning electron microscope (ZEISS, Oberkochen, Germany) to examine the surface structure and morphology of the samples, with an operating voltage of 15 kV. Transmission electron microscopy (TEM) characterization was carried out on a high-resolution transmission electron microscope (HRTEM, FEI F200X, Thermo Fisher Scientific, Hillsboro, OR, USA) to investigate the morphological structure and lattice spacing of the samples. In addition, energy-dispersive X-ray spectroscopy (EDS) (Oxford Instruments, Abingdon, UK) attached to the HRTEM was used to obtain the elemental composition, as well as line-scan profiles and elemental mapping distributions of various elements.”

The crystal structure was determined by XRD; “The crystal structure of the sample was determined using a Rigaku SmartLab X-ray diffractometer (Rigaku Corporation, Akishima, Japan) with Cu-Kα radiation (λ = 1.5406 Å) and an X-ray generator power of 3 kW. The measurements were performed over a 2θ range of 10° to 90° at a scan speed of 1°/min. Crystallite size was calculated using the Scherrer formula based on the data obtained from the test results.”

(3) $$D=\frac{k\lambda }{\beta COS\theta }$$

“In the formula, k = 0.943 is the shape factor of the crystallites, the X-ray wavelength λ = 0.154056 nm, β represents the full width at half maximum (FWHM) of the diffraction peak, and θ is the Bragg diffraction angle.”

The valence states of the elements were tested by XPS. Elemental electronic state analysis was conducted using the Thermo Fisher ESCALAB 250Xi X-ray photoelectron spectrometer (Thermo Fisher Scientific, Waltham, MA, USA). The test spectrum range was 100 eV, the narrow spectrum was 30 eV, the step size was 0.05 eV, and the dwell time was 40–50 ms. Charge correction was performed based on the binding energy of C1s = 284.80 eV. The fitting was carried out using the Avantage 5.9 software. Background type (Shirley, Tougaard) and fitting function (Gaussian–Lorentzian) were determined.

The specific surface areas and pore structures were determined by the N_2_ adsorption–desorption method. Using the ASAP 2460 instrument from Micromeritics (Norcross, GA, USA), 0.1 g of the sample to be tested was weighed and subjected to 120 °C, 6 h thermal desorption and cold desorption under vacuum conditions. Then, an adsorption–desorption test using liquid nitrogen was conducted.

The basic sites were determined by CO_2_-TPD characterization; The number of basic sites on the sample was determined using a Micromeritics AutoChem II 2920 chemisorption analyzer (Micromeritics, Norcross, GA, USA). The test conditions were as follows: approximately 50–100 mg of the sample was placed in a reactor tube and pretreated by heating from room temperature to 300 °C at a ramp rate of 10 °C/min under a helium flow, followed by purging with He for 1 h. After cooling to 50 °C, the sample was exposed to a CO_2_/He mixed gas for 1 h to reach saturation. The gas was then switched to pure He and purged for another 1 h to remove weakly adsorbed CO_2_ from the surface. Finally, the temperature was raised to 600 °C at 10 °C/min in a He atmosphere to carry out desorption, and the desorbed gases were detected using a thermal conductivity detector (TCD).

## 3. Results and Discussion

### 3.1. Characterization Analysis of Carbon Nitride Carrier

#### 3.1.1. Morphology Analysis

(1) Effect of hydrothermal synthesis temperature

**Figure 1:**
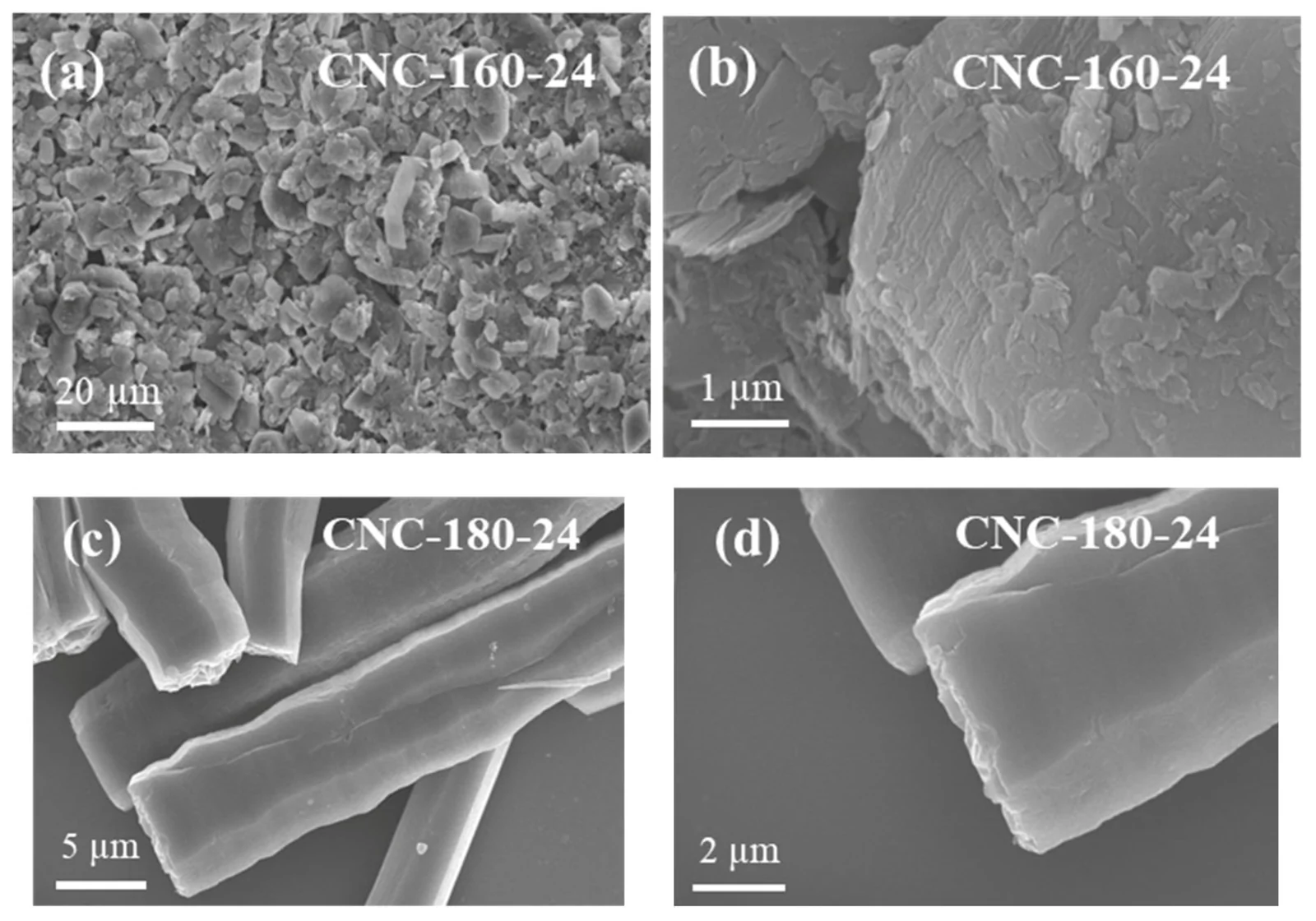
SEM images of carbon nitride precursors prepared at different hydrothermal temperatures. (**a**) CNC-160-24, 20 µm scale; (**b**) CNC-160-24, 1 µm scale; (**c**) CNC-180-24, 5 µm scale; (**d**) CNC-180-24, 2 µm scale.

Carbon nitride was prepared by a template-free hydrothermal method using melamine and urea as precursors. Figure 1 show that CNC-160-24 exhibits a bulk structure composed of multi-layer nitrogen–carbon composite nanosheets, and when the hydrothermal temperature is increased to 180 °C, the product is transformed into a structure with an obvious rod-like morphology (diameter > 1 μm). This morphological change may be due to the directional growth of the precursor along the longitudinal direction induced by water molecules through competitive hydrogen bonding at higher temperatures [17]. However, when the hydrothermal temperature rose to 200 °C, no obvious product was detected in the experiment, indicating that hydrothermal temperature has an important influence on the morphology evolution of the carbon nitride precursor.

(2) Effect of hydrothermal synthesis time

**Figure 2:**
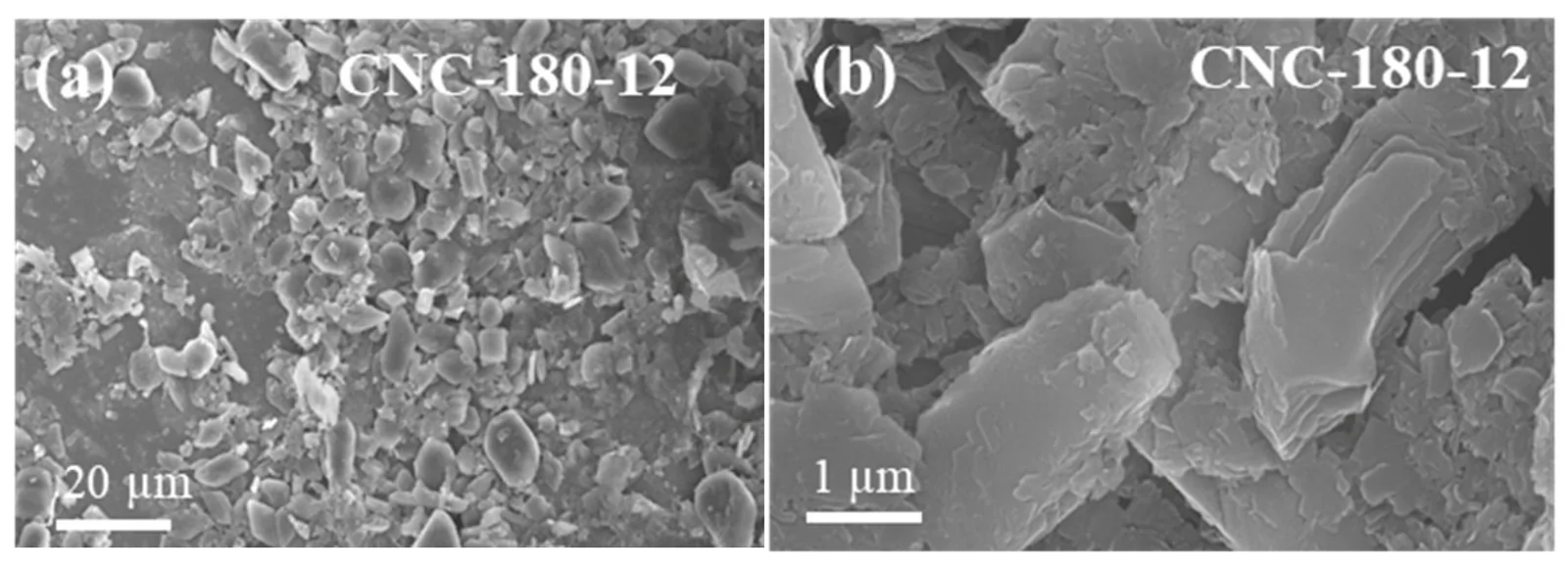
SEM images of carbon nitride precursor prepared by hydrothermal treatment at 180 °C for 12 h: (**a**) CNC-180-12, 20 µm scale; (**b**) CNC-180-12, 1 µm scale.

In order to reduce the energy consumption, the hydrothermal reaction time was shortened from 24 h to 12 h under the premise of keeping other experimental conditions unchanged. It can be seen in Figure 2 that the carbon nitride precursor prepared under this condition has an irregular block structure, and its morphological characteristics are quite different from those expected in this study. Considering the energy consumption and the structural characteristics of the product, the hydrothermal synthesis conditions were finally determined to be 180 °C and 24 h.

(3) Morphology and structure after calcination

**Figure 3:**
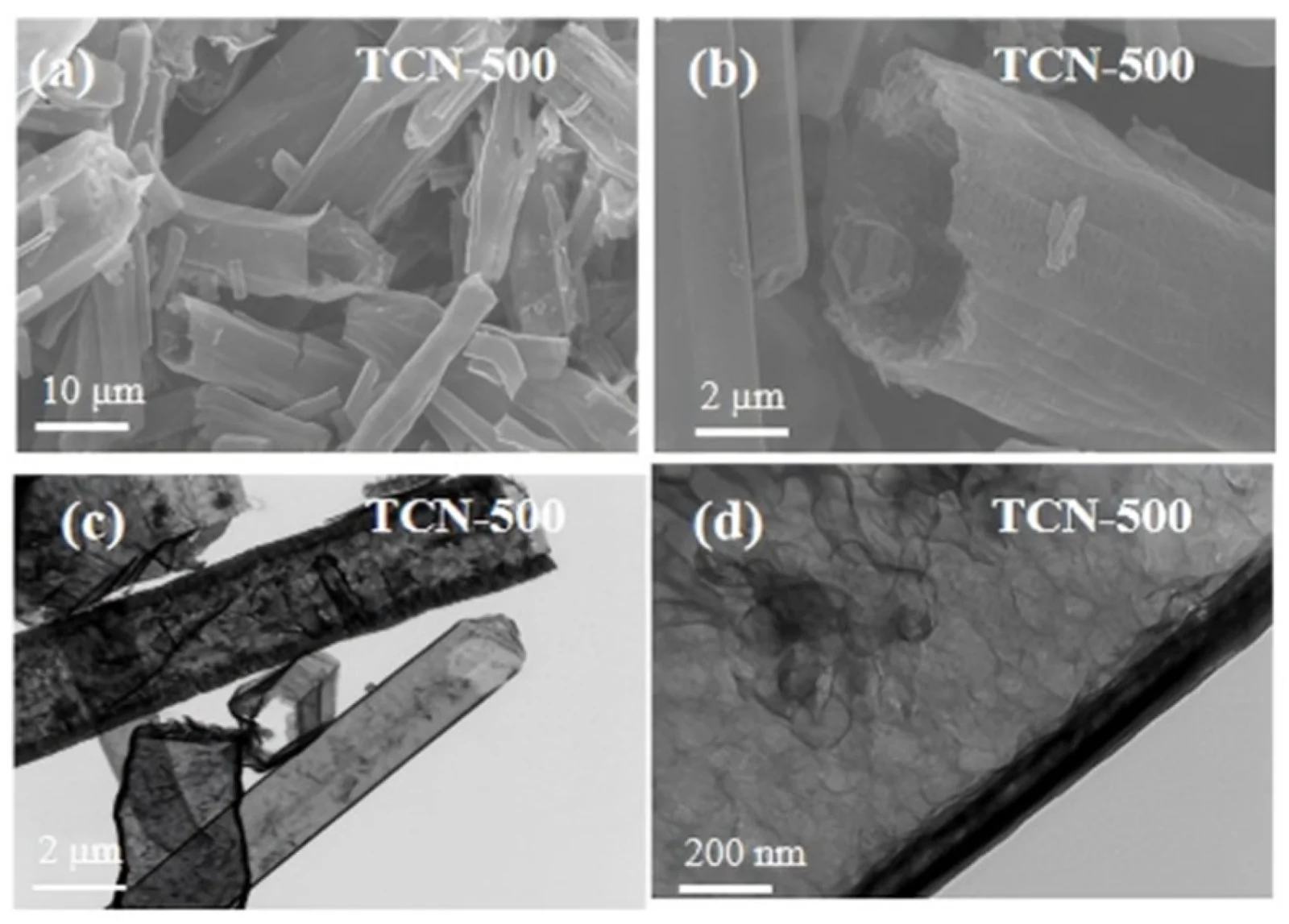
SEM images: (**a**) TCN-500, 10 µm scale; (**b**) TCN-500, 10 µm scale; TEM images: (**c**) TCN-500, 2 µm scale; (**d**) TCN-500, 200 nm scale.

Figure 3 is the SEM and TEM images of the product obtained by heating CNC-180–24 at 500 °C for 4 h in a N_2_ atmosphere. It can be seen from the figure that TCN-500 has a hollow tubular structure, and the length of the tube and the diameter of the nozzle are different. It can be seen from Figure 3d that there is flocculent material inside the hollow tube, which may be due to the incomplete conversion of the precursor during the condensation process. Overall, the calcination treatment has effectively transformed the rod-like precursor into a hollow tubular carbon nitride structure, which provides a favorable carrier basis for the subsequent loading of active metals.

#### 3.1.2. XRD Characterization Analysis

**Figure 4:**
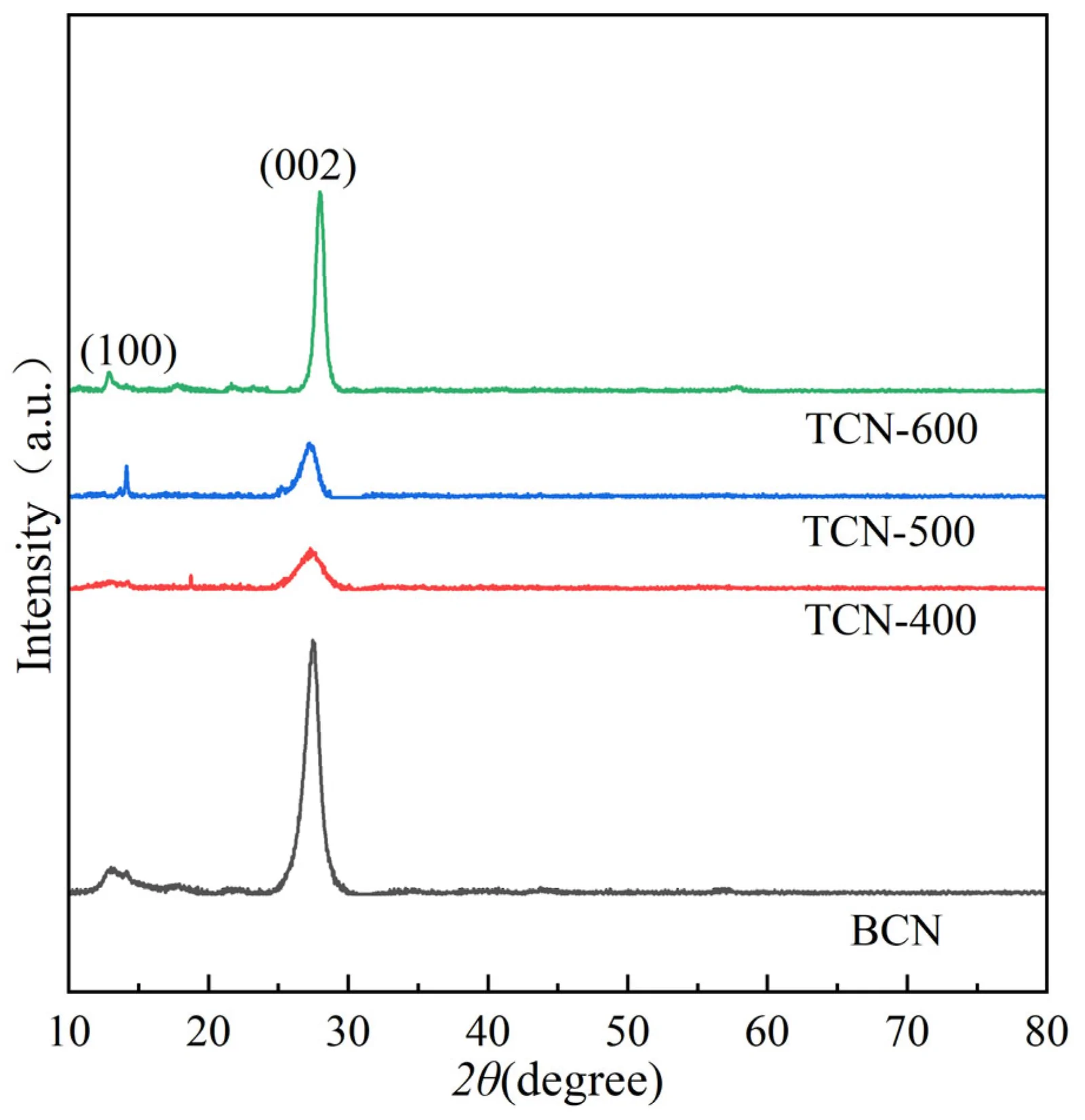
XRD diffraction patterns of different carbon nitride materials.

As shown in Figure 4, the characteristic diffraction peaks of the graphite phase structure appear at 13.2° and 27.4° for both tubular carbon nitride and bulk carbon nitride. Among them, the characteristic diffraction peak at 13.2° in the XRD pattern corresponds to the (100) crystal plane formed by the orderly stacking of triazine structural units [18], and the diffraction peak at 27.4° belongs to the (002) crystal plane, reflecting the interlayer stacking characteristics of the conjugated aromatic system [19]. With the increase in the calcination temperature from 400 °C to 600 °C, the (002) peak in the XRD has a high angle migration and shows an enhanced peak intensity, indicating that the interlayer spacing of the sample is further reduced and the stacking order between layers is improved. This change can be attributed to the interlayer spacing compression caused by high temperature-induced structural shrinkage and increased defects. The average grain sizes of the different carriers were calculated using the Scherrer method, and the size values are shown in Table 1. The largest grain is TCN-600, with a value of 11.4 nm. The smallest grain is TCN-400, with a value of 4.5 nm.

#### 3.1.3. XPS Characterization Analysis

**Figure 5:**
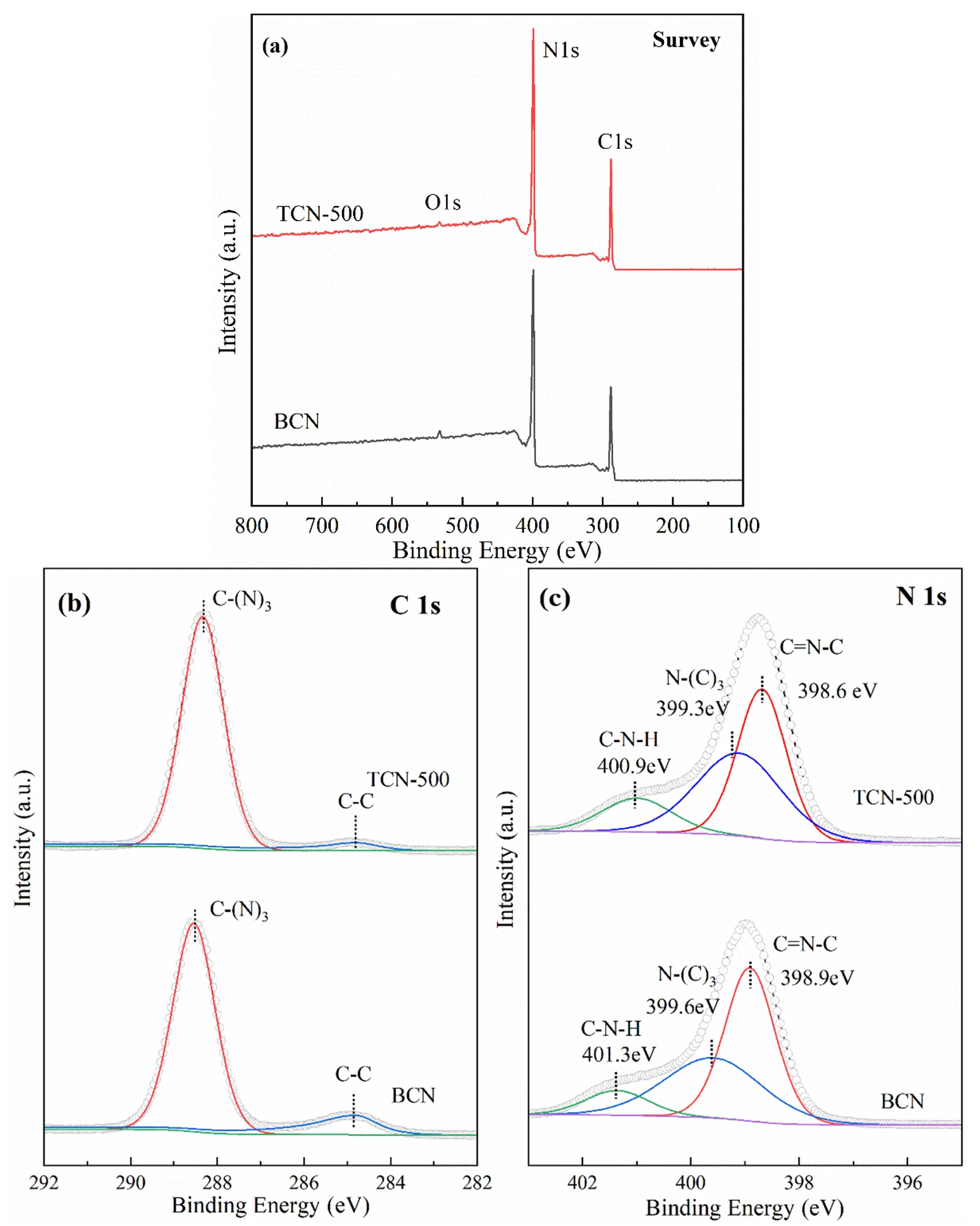
XPS spectra of TCN-500 and BCN: (**a**) survey; (**b**) C1s; (**c**) N1s.

Figure 5a is the full XPS spectra of TCN-500 and BCN. The high-resolution C1s spectrum of Figure 5b shows that the peak of BCN at 284.8 eV is a graphite C-C bond, and the peak at 288.3 eV belongs to an sp^2^ hybrid C-(N)_3_ group. The TCN-500 shifts about 0.2 eV to the high binding energy at the corresponding position and exhibits a lower N=C-N peak area (BCN: 87.26; TCN-500: 80.25). The results of the upward shift in the binding energy and the change in the N=C-N peak area show that the electron redistribution caused by carbon vacancy changes the local chemical environment [20]. As shown in Figure 5c, the N1s fine spectra of the BCN at 398.9 eV, 399.6 eV and 401.3 eV correspond to sp^2^ hybridized C=N-C, N-(C)_3_ and amino (C-N-H) structures, respectively [21,22,23]. In contrast, the above characteristic peaks in TCN-500 are shifted to the direction of low binding energy, and the C-N-H/N-(C)_3_ area ratio is increased from 0.27 of the BCN to 0.33. Among them, the area percentage of the amino (C-N-H) component of TCN-500 is 14.14%, which is higher than 10.32% of the BCN, indicating that the content of the functional nitrogen groups on the surface of the sample increased after calcination. These results indicate that the formation of carbon vacancies leads to the cleavage of CN heterocycles and the formation of more terminal amino groups, which changes the N coordination environment and electronic structure [24].

#### 3.1.4. N_2_ Adsorption–Desorption Analysis

**Figure 6:**
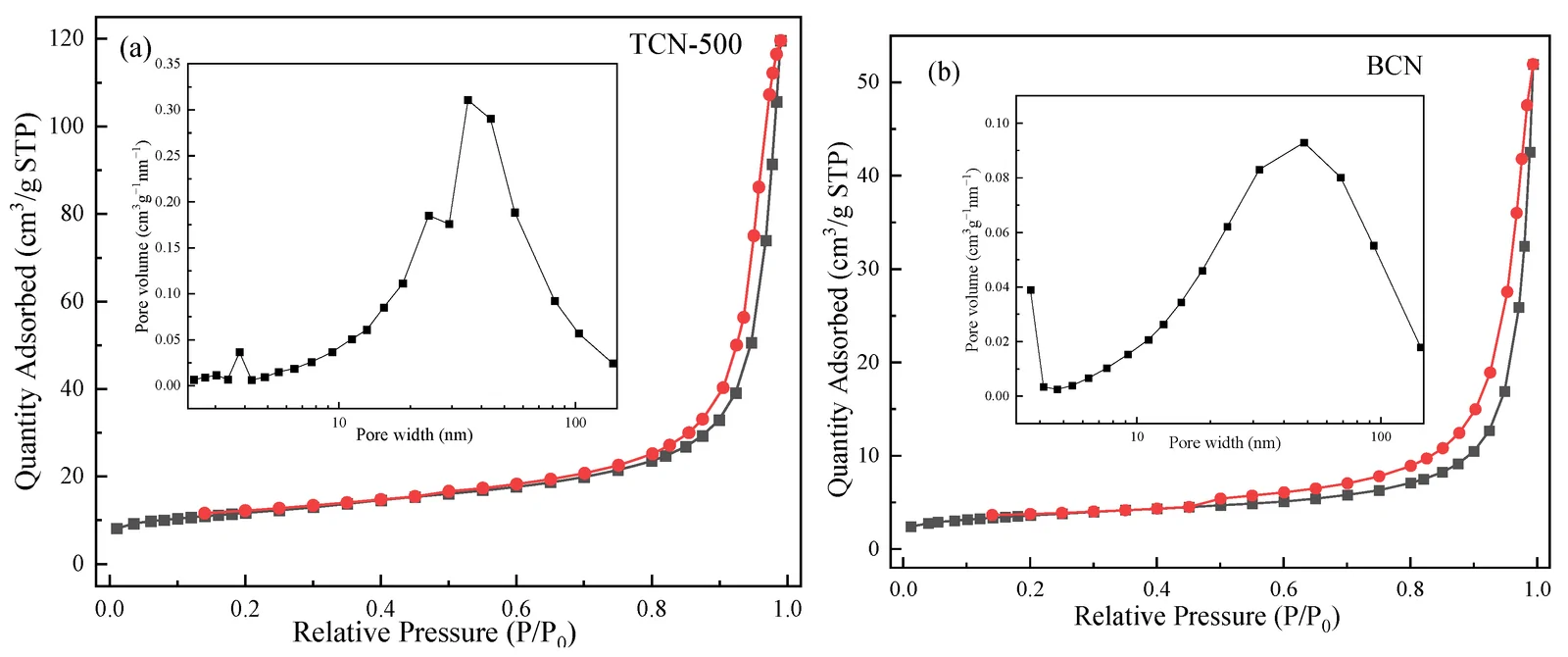
Nitrogen adsorption-desorption isotherms and pore size distribution (inset): (**a**) TCN-500; (**b**) BCN.

As shown in Figure 6, both TCN-500 and BCN exhibit typical type IV isotherm characteristics and have abundant mesoporous structures. Compared with the specific surface area of BCN (12.47 m^2^/g), the specific surface area of TCN-500 was significantly increased to 40.19 m^2^/g; the pore volume also increased from 0.0792 cm^3^/g to 0.1850 cm^3^/g. The larger specific surface area and pore volume can improve the adsorption performance of the catalyst and increase the contact probability between the reactant and the catalyst, thereby improving the catalytic performance.

### 3.2. Characterization Analysis of Supported Pd Catalyst

#### 3.2.1. Morphological Characteristics of Catalysts

**Figure 7:**
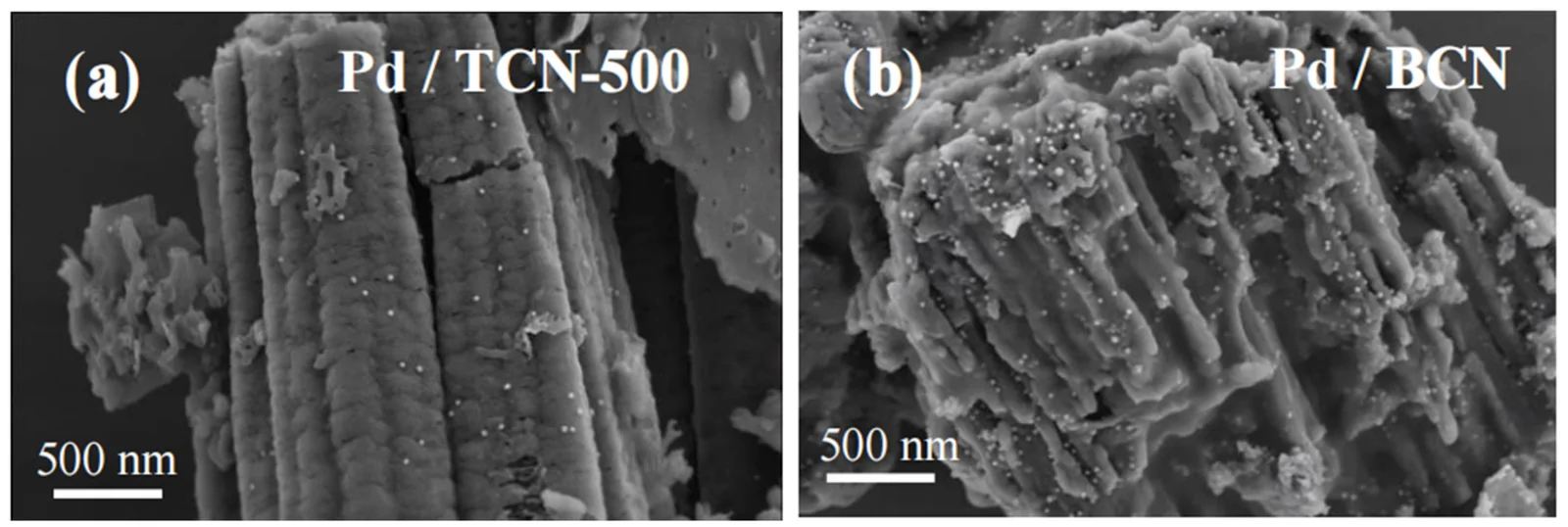
SEM images of catalysts: (**a**) Pd/TCN-500; (**b**) Pd/BCN.

Figure 7a shows that Pd/TCN-500 still presents the tubular morphology of TCN-500 after loading Pd, and Pd nanoparticles are mainly attached and distributed on the outer wall of the tubular structure. Figure 7b shows that Pd/BCN still presents a bulk structure after loading Pd, and the nanoparticles attached to its surface are denser than those on Pd/TCN-500. The above results show that the two carriers can maintain their respective skeleton structures during the loading process, and different morphologies and surface structures may lead to differences in the surface distribution of metal particles.

**Figure 8:**
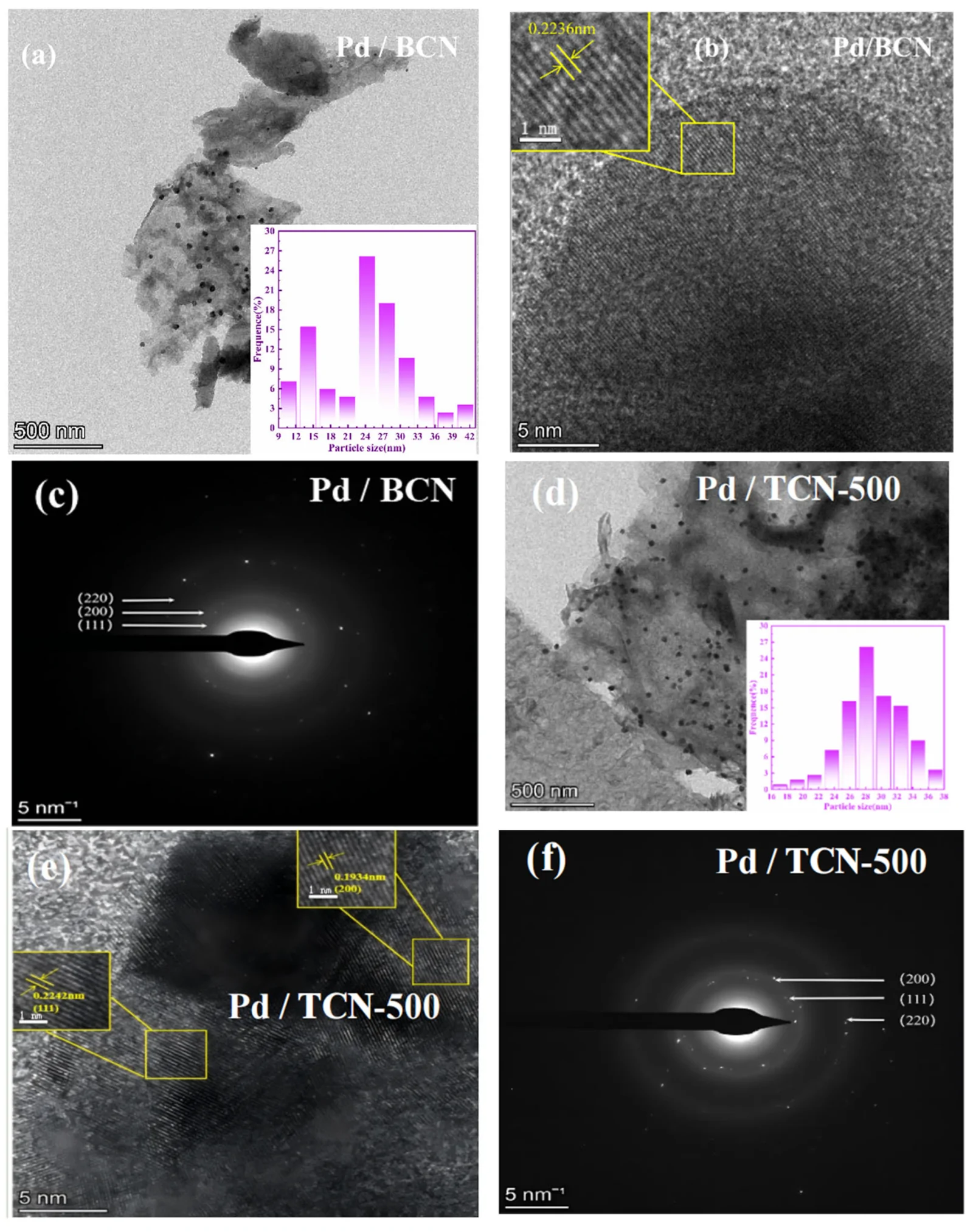
TEM (Illustration: Particle size distribution), HR-TEM and selected diffraction images: (**a**–**c**) Pd/BCN; (**d**–**f**) Pd/TCN-500.

Figure 8a shows that the Pd nanoparticles in Pd/BCN are mainly distributed on the surface of the BCN, which is consistent with SEM observation. The particle size distribution is relatively dispersed, below 42 nm, and shows a concentrated distribution in the range of about 14–31 nm; the average particle size is 26 ± 10.3 nm. The corresponding HR-TEM images (Figure 8b) distinguish clear lattice fringes, mainly exposing the (111) crystal plane of Pd; the crystal plane spacing is about 0.22 nm, and there are polycrystalline diffraction characteristics such as (200) and (220), which are consistent with the above XRD analysis results.

As shown in Figure 8d, the particle distribution in the local area of Pd/TCN-500 is more uniform, and the size is mainly concentrated at about 28 nm, while the average particle size is 27 ± 6.7 nm, which is close to the grain size estimated by XRD based on the (111) peak. Further HR-TEM observation showed that the crystal plane-spacing values corresponding to the lattice fringes were about 0.22 nm (111) and 0.19 nm (200), respectively, which was consistent with the XRD analysis results.

The TEM characterization of the two loading systems showed that the particle size distribution of Pd/BCN was wider and the uniformity was poor. In contrast, the Pd nanoparticles on Pd/TCN-500 have higher dispersion and more uniform size. This difference can be attributed to the selective adsorption and stable anchoring of Pd precursors by the abundant carbon vacancies in TCN-500 during the impregnation reduction process, as well as the spatial confinement effect of the tubular structure, thus promoting the uniform nucleation and growth of metal particles [25]. The above microstructure characteristics lay the structural foundation of carrier–metal synergy for the subsequent improvement of catalytic performance.

#### 3.2.2. XRD Analysis of the Catalyst

**Figure 9:**
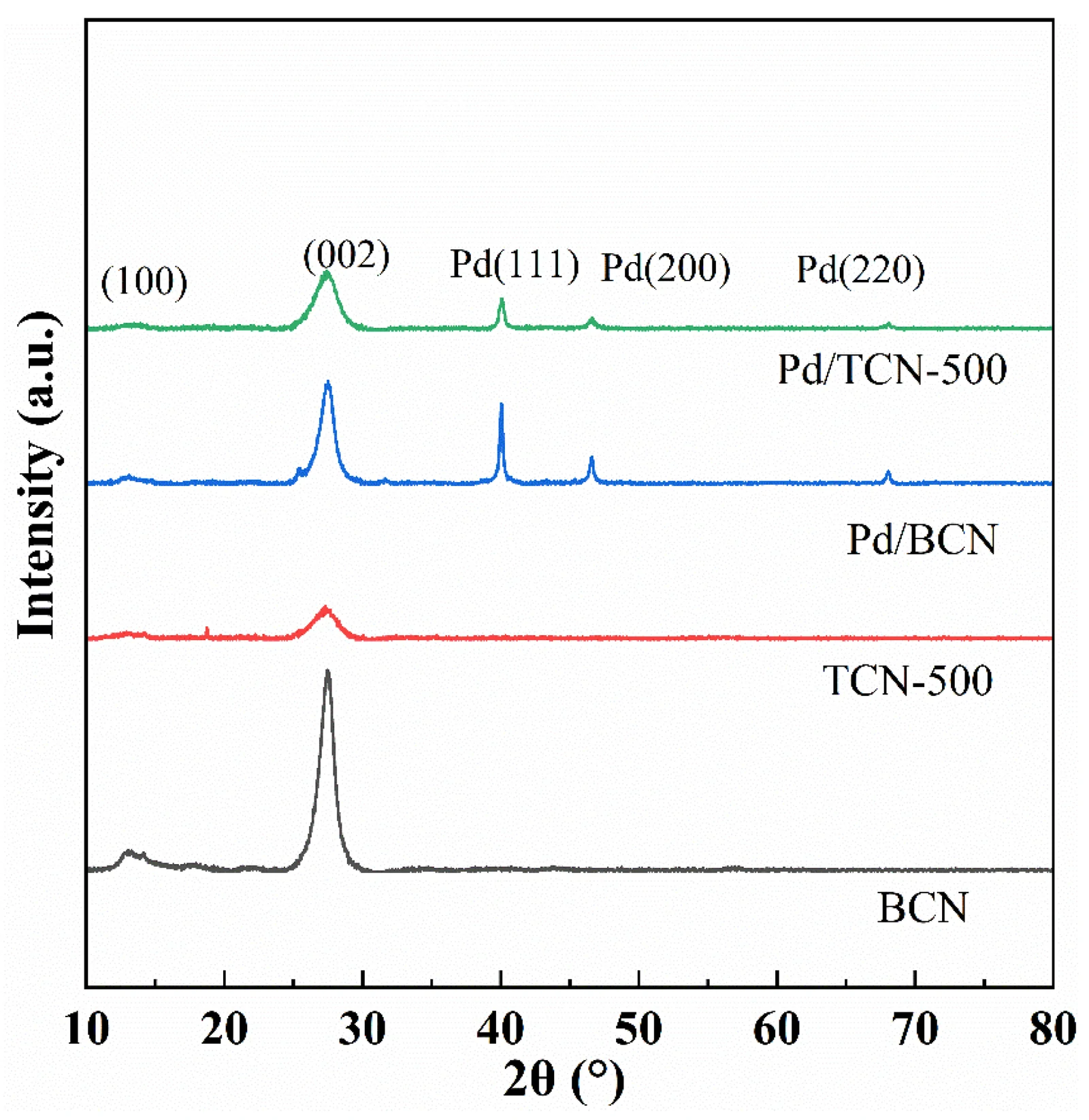
XRD patterns of different materials.

As shown in Figure 9, the XRD patterns of the BCN, Pd/BCN, TCN-500 and Pd/TCN-500 samples all show (100) diffraction peaks related to the orderly stacking of triazine units at 13.2° (d = 0.681 nm), as well as the (002) diffraction peaks originating from aromatic interlayer stacking at 27.4° (d = 0.326 nm). The above results show that the main skeleton of the carbon nitride carrier before and after Pd loading remains intact. At the same time, the (111), (200) and (220) diffraction peaks of the characteristic metal Pd appeared at 40.11°, 46.66° and 68.08° for Pd/BCN and Pd/TCN-500, respectively [26].As can be seen from Table 2, the particle sizes of Pd in catalysts Pd/BCN and Pd/TCN-500 were calculated based on XRD and were 32.2 nm and 19.0 nm, respectively. The particle size of the Pd is approximately the same as the value obtained from the TEM statistics. The results show that the ultrasonic-assisted impregnation method can effectively immobilize Pd nanoparticles on the surface of the support while maintaining the structural integrity of carbon nitride. In addition, the interfacial interaction between Pd and TCN-500 helps to inhibit particle agglomeration and improve metal dispersion and stability [27], which lays a good structural foundation for the improvement of catalytic performance.

#### 3.2.3. XPS Analysis of the Catalyst

**Figure 10:**
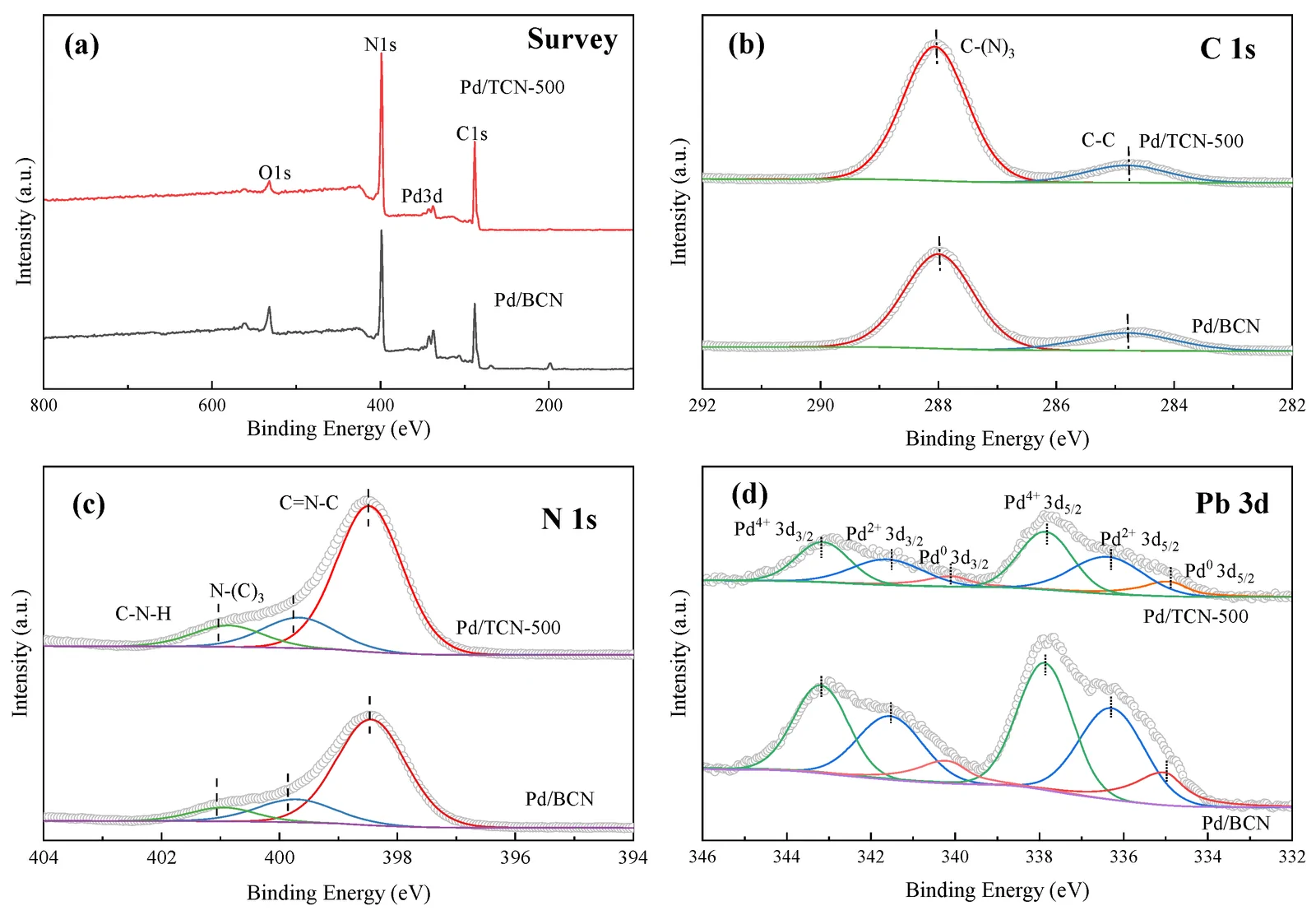
XPS spectra of Pd/TCN-500 and Pd/BCN: (**a**) survey; (**b**) C1s; (**c**) N1s; (**d**) Pd3d.

Figure 10a shows the full spectra of the Pd/BCN and Pd/TCN-500 XPS. The peaks near 288 eV, 398 eV and 336 eV appear to be the characteristic signals of C1s, N1s and Pd3d, respectively. Figure 10b is the high-resolution energy spectrum of C1s, in which the 284.8 eV peak corresponds to the C-C bond and the 288.0 eV peak corresponds to the sp^2^-hybridized N-C=N bond. Compared with the peak area ratio of the C-C bond to N-C=N in Pd/BCN (0.2461), the peak area ratio of Pd/TCN-500 increased to 0.2625, indicating that sp^2^ carbon sites in tubular carbon nitride carriers are more likely to form carbon vacancy defects. In addition, the N-C=N peak in Pd/TCN-500 shifts to a higher binding energy, indicating that the introduction of carbon vacancies leads to electron redistribution in the system and reduces the local electron density of the triazine ring. Figure 10c is the high-resolution energy spectrum of N1s, where C=N-C (sp^2^), N-(C) _3_ and amino (C-N-H) were observed at 398.4 eV, 399.7 eV and 400.9 eV, respectively. In addition, compared to Pd/BCN, the area percentage of the C-N-H group in Pd/TCN-500 increased from 9.0% to 11.25% (see Table 3), and the C=N-C in Pd/TCN-500 shifted to a higher binding energy. Combined with the change in C1s, it can be inferred that the formation of carbon vacancies promotes the partial cleavage of nitrogen-containing heterocycles and the formation of more terminal amino groups, which in turn changes the local coordination environment and electronic structure.

In Figure 10d, the Pd 3d of Pd/TCN-500 is fitted into four peaks: Pd^0^ 3d_3/2_ and Pd^0^ 3d_5/2_ are located at 336.6 and 341.8 eV, respectively; and Pd^2+^ 3d^3/2^ and Pd^2+^ 3d_5/2_ are located at 335.1 and 340.3 eV, respectively. The Pd^0^ component exhibits a relatively low binding energy, which is usually related to the electron donor effect at the support–metal interface, indicating that there is an effective interfacial electron coupling between the TCN-500 and Pd nanoparticles. Based on the quantitative fitting results of Pd 3d (see Table 3), the proportion of Pd^0^ in Pd/TCN-500 is slightly higher than that of Pd/BCN, while the relative content of high-valence Pd (Pd^2+^) is not much different. The above valence distribution shows that the TCN-500 support is helpful to stabilize the electron-rich metal Pd species and maintain a certain proportion of high-valence Pd, which provides conditions for the possible valence cycle during the reaction.

#### 3.2.4. Analysis of Catalysts by CO_2_-TPD

**Figure 11:**
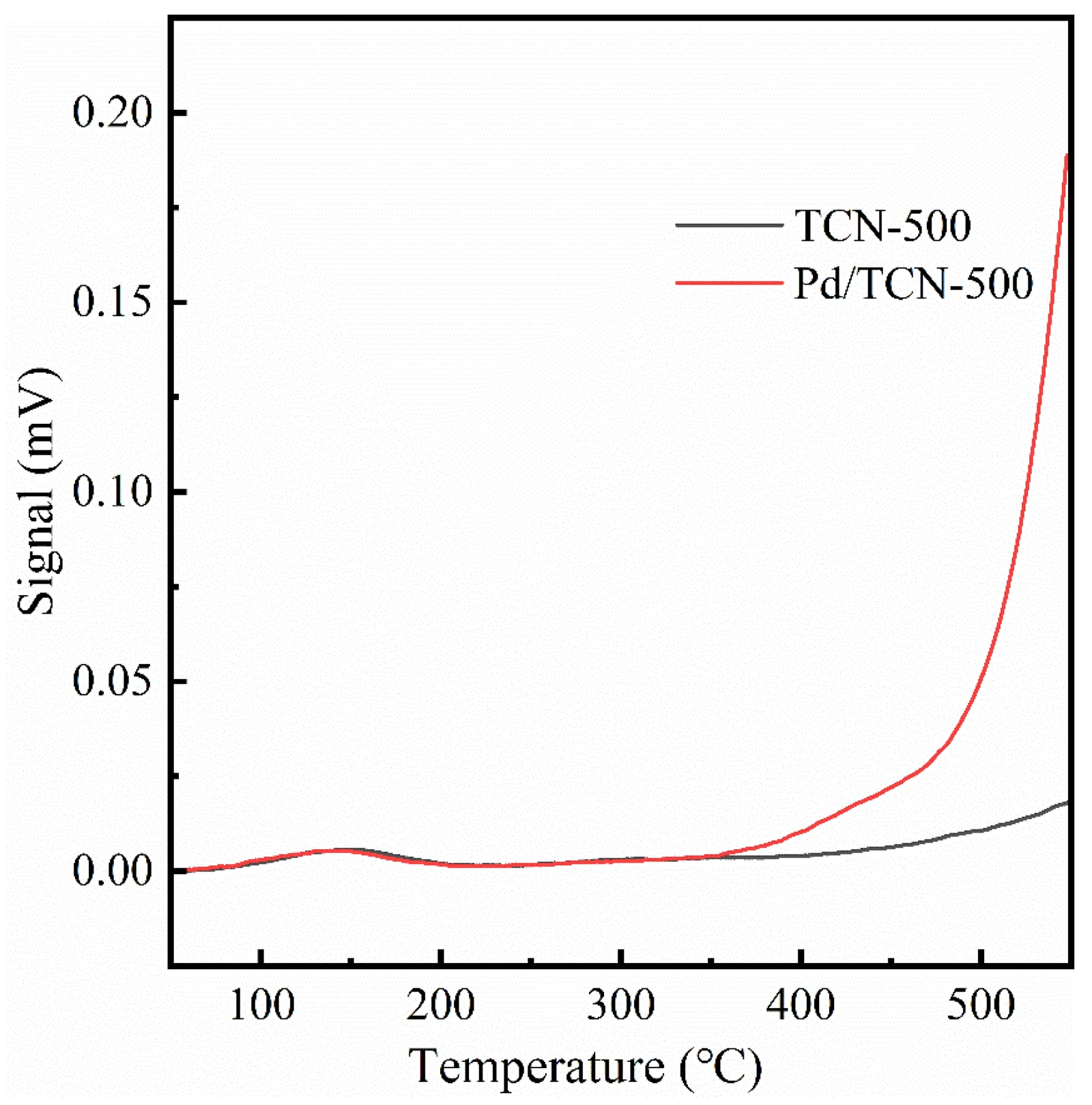
CO_2_-TPD spectra of different catalysts.

As shown in Figure 11, the CO_2_-TPD curve of the sample mainly presents two desorption peaks: a low-temperature peak of about 145 °C and a high-temperature peak in the range of 450–550 °C. The former is usually related to the physical/weak chemical adsorption of acidic CO_2_ by weak basic sites, while the latter corresponds to the strong adsorption of CO_2_ by strong Lewis basic sites and the stable binding of carbonate/bicarbonate species. The quantitative results of various alkaline sites are shown in Table 4. It can be seen from the test that the total alkali amount of TCN-500 is 0.4866 mmol/g, while the total alkali amount of Pd/TCN-500 is significantly increased to 1.7172 mmol/g, and the proportion of desorption contribution in the high-temperature region is significantly increased. The results show that after the introduction of the Pd nanoparticles, the surface/edge defects and electron-rich regions of the carrier are further increased and activated, which enhances the strength and number of Lewis base centers such as adjacent nitrogen-containing sites, thereby facilitating the dispersion and anchoring of metal species and providing more effective sites for substrate activation and intermediate stability in the subsequent reaction process [28].

### 3.3. Catalyst Activity Test

**Figure 12:**
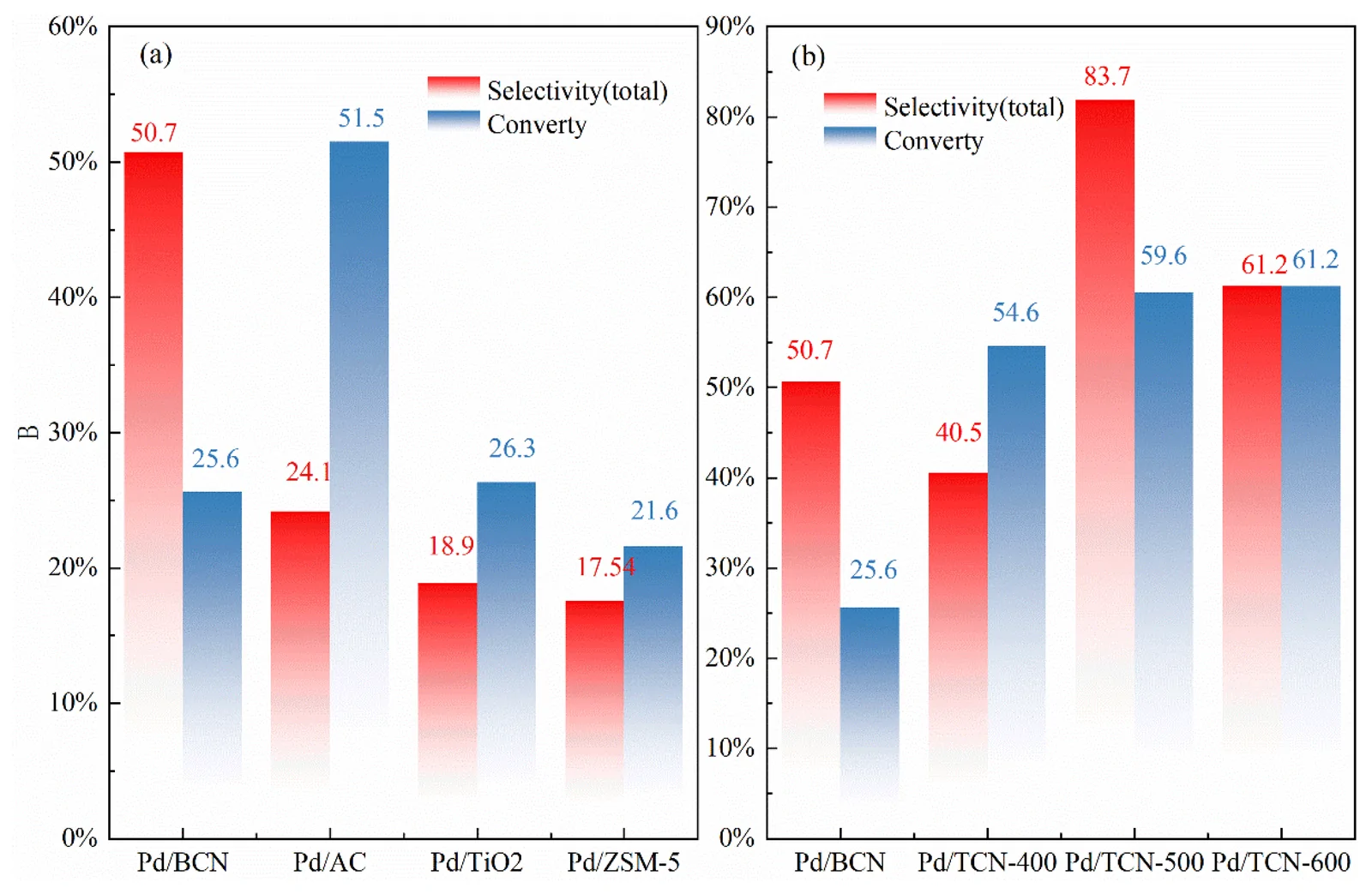
Catalytic performances of different catalysts: (**a**) test of catalysts with different supports; (**b**) test of carbon nitride supported catalyst.

Figure 12a shows that when BCN is used as the carrier, the selectivity of the target product of acetylene dicarbonylation is up to 50.7%. Although higher acetylene conversion can be obtained when activated carbon (AC) is used as the carrier, the selectivity of the target product is significantly lower than that of Pd/BCN. Figure 12b shows the catalytic performances of ordinary carbon nitride and tubular carbon nitride, obtained at different calcination temperatures, as carriers; the tubular carbon nitride formed at 400 °C has low selectivity, which is speculated to be related to the low calcination temperature, which has led to insufficient formation of pore structure and carbon defects, and it is difficult to provide an excellent loading environment for metal active sites. Although the conversion of Pd/TCN-500 is similar to that of Pd/TCN-600, the total selectivity of Pd/TCN-500 is the highest (83.7%). Further calcination at 600 °C may cause structural rearrangement/shrinkage and other changes, resulting in a decrease in selectivity.

Combined with the above multiple characterization results, the structure–performance relationship can be further established. Firstly, 500 °C treatment is conducive to the formation of more carbon vacancies and nitrogen-containing functional sites, as well as the improvement of Lewis basicity and specific surface area/pore volume, thus providing richer and stronger metal anchoring sites and substrate adsorption/activation sites. The XPS and CO_2_-TPD results support the above judgment. Secondly, the spatial confinement and surface defects of the tubular structure promote the stability and uniform dispersion of Pd species. The TEM and XRD results show that the distribution of metal particles on Pd/TCN-500 is more uniform and the characteristics of the exposed (111) crystal plane are more significant. The synergistic effect of the above factors makes Pd/TCN-500 have both high selectivity and good activity in the acetylene double carbonylation reaction, which is better than other carrier systems.

### 3.4. Catalyst Life Test

**Figure 13:**
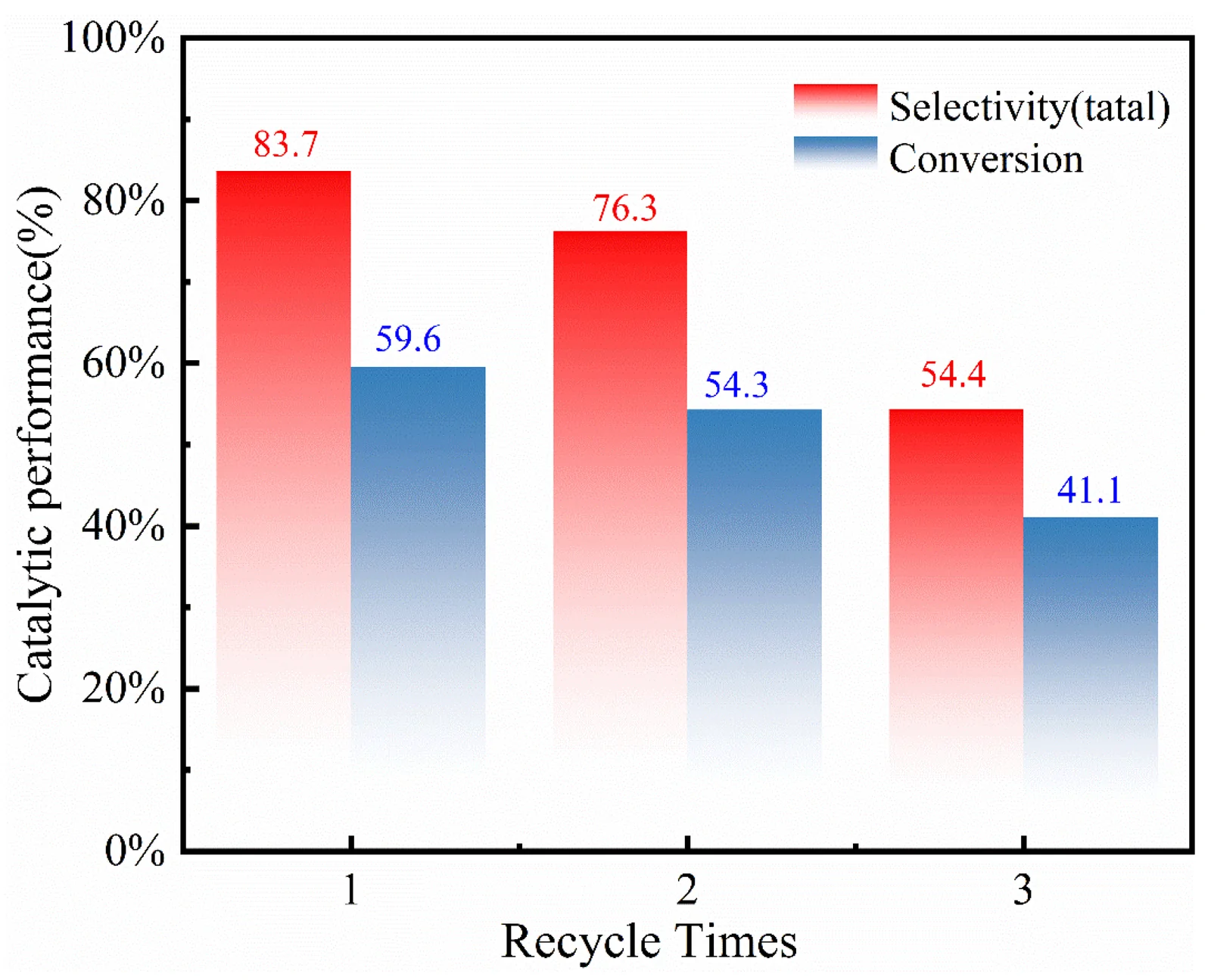
Cyclic stability of Pd/TCN-500.

The cycle evaluation was carried out with the Pd/TCN-500 with the best selectivity. Figure 13, shows that with the increase in the number of cycles, the selectivity of the target product decreased from 83.7% to 54.4%, and the conversion of acetylene also decreased to 41.1%. The activity and selectivity of the catalyst decreased significantly after three cycles, which may have been due to the deterioration of the carrier structure and the passivation of the defect sites. On the one hand, the presence of CO, O_2_/air and I-species in the reaction system may induce the slow oxidation, fracture or agglomeration of the g-C_3_N_4_ nanotube skeleton under a certain temperature and pressure for a long time, resulting in the destruction of the original regular tubular/porous structure and decreases in specific surface area and pore structure integrity. Previous work has shown that strong oxidation treatment can significantly weaken the catalytic activity of g-C_3_N_4_ [29], indicating that its surface-reducing sites are crucial for maintaining catalytic performance. On the other hand, the coordination N sites and carbon vacancies enriched on the surface of TCN-500 are the key defect centers for anchoring Pd nanoparticles and regulating their electronic structure in the initial state. Related studies have shown that N or C vacancies in g-C_3_N_4_ can induce skeleton electron depletion and significantly enhance metal–support interaction, thereby improving the dispersion and stability of metal species [30]. During multiple cycles, these defects/functional sites may be oxidized or occupied by strongly adsorbed species, on one hand, and their local electronic structures may also undergo irreversible changes, resulting in weakened bonding strength at the Pd-N/defect interface and simultaneous deterioration of the number and properties of the active centers that can participate in the Pd^0^/Pd^2+^ cycle. In summary, it is speculated that the chronic oxidative damage to the g-C_3_N_4_ nanotube framework and the gradual passivation of defect sites may jointly contribute to the significant decline in the catalytic performance of the Pd/TCN-500 catalyst after repeated use.

## 4. Conclusions

In this study, tubular carbon nitride (TCN) was prepared by hydrothermal calcination, and Pd was loaded on it. A new route for the design of an acetylene double carbonylation heterogeneous catalyst with “morphology control-defect engineering-metal/carrier electron coupling” was proposed. In order to highlight the regulation of carrier morphology and defect structure on Pd dispersion and product selectivity, the nominal loading of Pd in each catalyst was uniformly controlled to 5 wt %. The structure–performance differences of different carrier systems were systematically compared under the same amounts of precious metals. The effects of Pd loading on dispersion and selectivity were not further studied. The related optimization will be further studied as the focus of follow-up work. Compared to traditional bulk g-C_3_N_4_, TCN-500 has a higher specific surface area and larger pore volume, which provides more stable anchoring sites and more favorable mass transfer channels for Pd nanoparticles. After the introduction of Pd, the total base amount and strong Lewis-base center were significantly increased, indicating that the carbon vacancies and nitrogen-containing sites were effectively activated, thereby enhancing the metal–support coordination ability and substrate activation ability. At the same time, Pd is highly dispersed on TCN-500 and preferentially exposes the (111) crystal plane, and the interface–electron interaction is stronger, which synergistically improves the adsorption and conversion efficiencies of key intermediates so that the acetylene double carbonylation reaction exhibits higher selectivity and better comprehensive catalytic performance. Providing an excellent catalyst support for the acetylene dicarbonylation reaction offers a novel perspective.

## Figures and Tables

**Table 1:** Average grain sizes of different carriers.

Support Name	Crystallite Size (D/nm)
BCN	6.9
TCN-400	6.5
TCN-500	4.5
TCN-600	11.4

**Table 2:** The grain sizes of Pd (111) crystals in the catalyst before the reaction.

Catalyst	Crystallite Size (D/nm)
Pd/BCN	32.2
Pd/TCN-500	19.0

**Table 3:** The peak area data of different structures of Pd/BCN and Pd/TCN-500.

Element	Chemical Bond	Pd/BCN at.%	Pd/TCN-500 at.%
C1s	C-C	19.75	20.79
	N-C=N	80.25	79.21
N1s	C=N-C	73.2	72.09
	N-(C)_3_	17.8	16.66
	C-N-H	9.0	11.25
Pd 3d	Pd^0^	39.43	40.74
	Pd-O(Pd^2+^)	60.57	59.26

**Table 4:** Alkaline scales of TCN-500 and Pd/TCN-500.

Catalyst	Alkali Content (mmol/g)		
	Weakly Basic Site	Strongly Basic Site	Total Base Content
TCN-500	0.1275	0.3591	0.4866
Pd/TCN-500	0.1008	1.6164	1.7172

## Data Availability

The original contributions presented in this study are included in the article. Further inquiries can be directed to the corresponding author.
